# Deltoid muscle volume affects clinical outcome of reverse total shoulder arthroplasty in patients with cuff tear arthropathy or irreparable cuff tears

**DOI:** 10.1371/journal.pone.0174361

**Published:** 2017-03-29

**Authors:** Jong Pil Yoon, Anna Seo, Jeong Jun Kim, Chang-Hwa Lee, Seung-Hun Baek, Shin Yoon Kim, Eun Taek Jeong, Kyung-Soo Oh, Seok Won Chung

**Affiliations:** 1 Department of Orthopaedic Surgery, Kyungpook National University College of Medicine, Daegu, Korea; 2 Institute of Advanced Convergence Technology, Kyungpook National University, Daegu, Korea; 3 Department of Orthopaedic Surgery, Konkuk University School of Medicine, Seoul, Korea; Harvard Medical School/BIDMC, UNITED STATES

## Abstract

We aimed to estimate the interrelation between preoperative deltoid muscle status by measuring the 3-dimensional deltoid muscle volume and postoperative functional outcomes after reverse total shoulder arthroplasty(RTSA). Thirty-five patients who underwent RTSA participated in this study. All patients underwent preoperative magnetic resonance imaging(MRI) as well as pre- and postoperative radiography and various functional outcome evaluations at least 1 year. The primary outcome parameter was set as age- and sex-matched Constant scores. The 3-dimensional deltoid muscle model was generated using a medical image processing software and in-house code, and the deltoid muscle volume was calculated automatically. Various clinical and radiographic factors comprising the deltoid muscle volume adjusted for body mass index(BMI) were analyzed, and their interrelation with the outcome parameters was appraised using a multivariate analysis. As a result, all practical consequences considerably improved following surgery(all p<0.01). Overall, 20 and 15 indicated a higher and a lower practical consequence than the average, respectively, which was assessed by the matched Constant scores. The deltoid muscle volume adjusted for BMI(p = 0.009), absence of a subscapularis complete tear (p = 0.040), and greater change in acromion-deltoid tuberosity distance(p = 0.013) were associated with higher matched Constant scores. Multivariate analysis indicated that the deltoid muscle volume was the single independent prognostic factor for practical consequences(p = 0.011). In conclusion, the preoperative deltoid muscle volume significantly affected the functional outcome following RTSA in patients with cuff tear arthropathy or irreparable cuff tears. Therefore, more attention should be paid to patients with severe atrophied deltoid muscle who are at a high risk for poor practical consequences subsequent to RTSA.

## Introduction

Reverse total shoulder arthroplasty (RTSA) is a useful treatment option for patients with irreparable massive rotator cuff tears and cuff tear arthropathy (CTA).[1,2,3,4] Even though recently a less medialized design of reverse shoulder prosthesis is gaining popularity, the RTSA is grounded on the Grammont concept of fixed fulcrum prosthesis, medialization and lowering of the center of glenohumeral rotation. [1,5] The goal of RTSA is to compensate the dysfunctional rotator cuff after CTA and to improve a stability of shoulder joint, thus allowing elevation of the upper extremity with alternative function of the deltoid muscle.[6]

Following RTSA, the shoulder kinematics is altered as the deltoid becomes the primary motor muscle for flexion and abduction, and its muscle power is vital for shoulder motion. [6] Several authors indicated that muscle power is related to muscle volume, [7], [8] and muscle volume is directly associated with muscle function. [8], [9], [10], [11]

Even if the deltoid muscle has played an important role in the smooth operation of reverse prosthesis, only a few studies have measured deltoid volume and evaluated the correlation between deltoid volume and postoperative function subsequent to RTSA. Age [12], obesity [13], status of the remained rotator cuff muscle [14,15], scapular notching [16,17], and lengthening of the lever arm[18] have been considered essential factors for clinical consequences subsequent to RTSA.

This study aimed to appraise the correlation between preoperative deltoid muscle volume and postoperative practical consequences subsequent to RTSA by panoptically analyzing the demographic, clinical, and radiologic factors involving known prognostic factors and preoperative deltoid volume. Patients who underwent RTSA were included in the analysis. We hypothesized that the deltoid muscle volume is a vital prognostic factor for postoperative practical consequences subsequent to RTSA.

## Materials and methods

### Patients

The study protocol was approved by Konkuk University Medical Center Institutional Review Board (IRB no. KUH1060101). The need for consent was waived by the ethics committee of the author’s institute. All clinical data were prospectively collected in our database and retrospectively reviewed.

Among the 58 patients who underwent RTSA between June 2010 and October 2014 at our institute, we appraised 43 patients who underwent RTSA only for CTA or irreparable rotator cuff tears. Eight patients were lost to follow-up before the first postoperative year; the remaining 35 patients with sufficient follow-up (CTA = 25, irreparable rotator cuff tear = 10) were included in the investigation. Persistently severe symptoms, difficulty in fulfilling daily activities with a constant decreased shoulder motion in physical examinations, and no improvement in >6 months of conservative treatments were regarded as indications for RTSA. All patients underwent preoperative magnetic resonance imaging (MRI) and preoperative and 1 year postoperative radiograph positions. They were appraised by standardized patient and physician reported outcome measurements comprising the visual analog scale (VAS) for evaluating pain, simple shoulder test (SST), Constant score, American shoulder and elbow surgeons (ASES) score, and range of motion (ROM) preoperatively and at least 1 year postoperatively. All patients had immerse rotator cuff tears, and those with criteria grade 4 or 5 by Hamada [19] were regarded as having CTA. Exclusive criteria comprised salvage operation for failure after prior arthroplasty (n = 1) or after open reduction and internal fixation after proximal humerus fracture and dislocation (n = 4) or infective arthritis (n = 3), acute proximal humerus fracture and dislocation (n = 5), systemic arthropathies (n = 2), and less than minimal (1 year) follow-up (n = 8). The patients comprised 8 men and 27 women with a mean age of 74.77±4.23 (range, 66–84) years at the time of surgery. The mean follow-up period was 16.5±5.94 (range, 12–35) months.

The dominant shoulder was impacted in 29 patients. Demographic data are listed in Table 1.

**Table 1:** Demographic and radiographic data.

Variables	Data
Age (years)	74.77±4.23 (range, 66–84)
Gender (n)	Male, 8; Female, 27
Symptom duration (months)	54.17±73.90 (range, 6–240)
Etiology (n)	CTA, 25; irreparable massive tear, 10
Side of involvement (n)	Dominant, 29; Non-dominant, 6
Body mass index (kg/m^2^)	25.67±2.87 (range, 20.28–32.53)
Diabetes (n)	Yes, 10; No, 25
Hypertension or any heart disease (n)	Yes, 16; No, 19
Bone mineral density	-1.99±1.30 (range, -5.20–0.50)
Shoulder usage level (n)	High, 9; medium, 11; low, 15
Subscapularis integrity (n)	Intact or partial tear, 25; complete tear, 10
Pseudoparalysis (n)	Yes, 29; No, 6
Fatty infiltration of the supraspinatus*	3.05±0.90
Fatty infiltration of the infraspinatus	2.88±1.05
Fatty infiltration of the subscapularis	2.17±1.20
Fatty infiltration of the teres minor	1.74±1.26
Volume of the total deltoid (mm^2^)/BMI (kg/m^3^)	3803.55±1792.51
Massive tear grade by Hamada classification (n) ^†^	G1, 4; G2, 10; G3, 3; G4a, 8; G4b, 7; G5, 3
Postoperative notching (n)^‡^	Yes, 20; No, 15
Notching grade by Sirveaux classification	G0, 15; G1, 11; G2, 7; G3, 2; G4, 0
Inferior overhang (n) (mm)	Yes, 31; No, 4; mean, 5.06±3.04
Glenosphere version (°)	100.13±10.31
Glenosphere-scapular neck angle (°)	94.87±11.06
Preoperative acromion-DT distance (mm)	136.16±13.65
Postoperative acromion-DT distance (mm)	163.48±14.11
Change of the acromion-DT distance (mm)	27.03±12.10
Preoperative COR distance (mm)	18.54±6.31
Postoperative COR distance (mm)	39.07±6.76
Change of the COR distance (mm)	20.52±5.51

*Fatty infiltration was graded according to the criteria by Goutallier et al.[22]

^†^Massive rotator cuff tear was graded according to the criteria by Hamada et al.[19]

^‡^Scapular notching was graded according to the criteria by Sirveaux et al.[24]

CTA, cuff tear arthropathy; BMI, body mass index; DT, deltoid tuberosity; COR, center of rotation.

### Evaluation of factors involved in the surgical outcomes

Multiple factors that may affect RTSA consequences were prospectively collected, measured, and evaluated (Table 1). These constituent elements comprised age, sex, symptom duration, etiology, side of involvement, body mass index (BMI), integrity of the subscapularis, concomitant medical disease, shoulder usage level, existence of pseudoparalysis, status of each rotator cuff muscle, massive cuff tear grade by Hamada classification, [19] and various radiologic parameters, as well as the volume of the deltoid muscle.

Bone mineral density (BMD) was measured using dual energy X-ray absorptiometry at the last outpatient visit before operation. The lowest T-score of the proximal femur and lumbar spine was recorded with the exception of the value for the Ward’s area of the proximal femur.

For shoulder usage level, high level was described as involved in dynamic sports and manual labor, medium level as work with static sports and less activity, and low level as rarely participating in sports and retired. In this study, relatively high proportion of patients (9/35, 25.7%) showed a high shoulder usage level. Even though they did not play strenuous sports, all of them were manual labors (7 farmers and 2 cleaners). Pseudoparalysis was described as shoulder forward flexion <90° in the presence of full passive anterior flexion as a previous study suggested [20]. Fatty degeneration (FD) of the rotator cuff muscle, including the supraspinatus, infraspinatus, and subscapularis was assessed using the preoperative MRI at the most lateral section of the oblique sagittal image, while the scapular spine was still continuous with the body of the scapula forming a Y-shape.[21] This evaluation was carried out in accordance with the criteria by Goutallier et al. [22] and Fuchs et al. [23]

**Fig 1:**
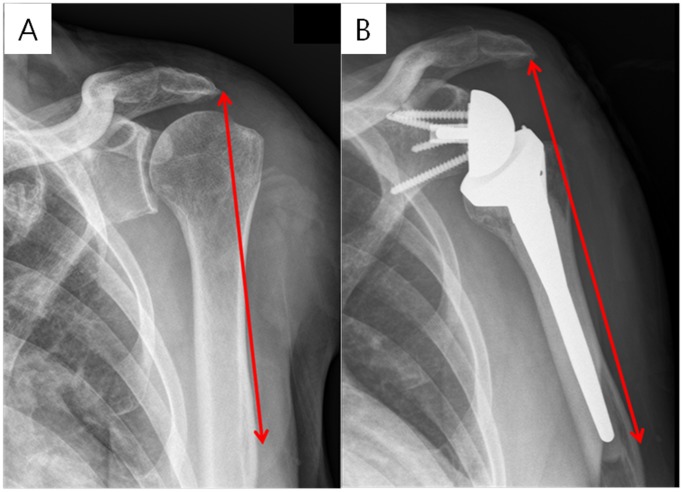
The measurement of the acromion-deltoid tuberosity distance. The (A) preoperative and (B) postoperative measurements are shown.

Radiographic appraisals were made using the standard AP radiographs with specific positions (the arm in neutral rotation and 0° abduction), as well as axial radiographs, at the final follow-up (>1 year) for scapular notching, inferior overhang of the glenosphere, glenosphere version, glenosphere-scapular neck angle, change in the acromion-deltoid tuberosity distance after operation, and center of rotation (COR) medialization. Scapular notching was categorized under the classification system by Nerot-Sirveaux [24] on AP radiographs. Inferior glenosphere overhang was measured on AP radiographs as the direct distance between the inferior margin of the glenosphere and inferior glenoid neck. [25] Glenosphere version was measured on the axial radiographs as the angle formed by a line connecting the most anterior medial and posterior medial aspects of the glenosphere and a line connecting the midpoint of the glenoid with the most medial part of the scapular spine. [26] Glenosphere-scapular neck angle was decided by 2 lines (a line connecting the most proximal medial and distal medial aspects of the glenosphere and a line along the inferior lateral bone of the glenoid rim) on the AP radiographs. [25] Moreover, the acromion-deltoid tuberosity distance was described as the length between the acromion and the deltoid tuberosity of the humerus with the arm in neutral rotation and 0° abduction as described by a previous study [27]. (Fig 1)

The COR of the glenohumeral joint and COR distance were determined as reported by a previous study [28]. The postoperative elevated or medialized length of each measurement was computed. Moreover, the correlation between these radiographic parameters as well as diverse demographic and preoperative constituent elements and the clinical consequences were appraised.

### Measurement of the deltoid volume

**Fig 2:**
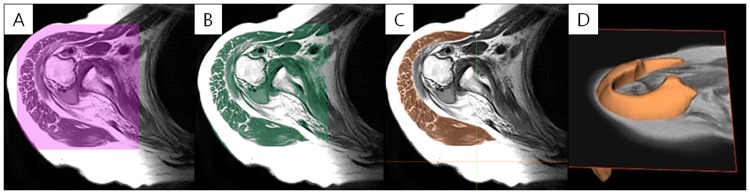
Image processing. (A) Designation of the region of interest (ROI) including the entire deltoid muscle. (B) Separation of the bone and fat areas from the established ROI. (C) Demarcation of the deltoid muscle area. (D) Generation of the 3-dimensional deltoid muscle model.

**Fig 3:**
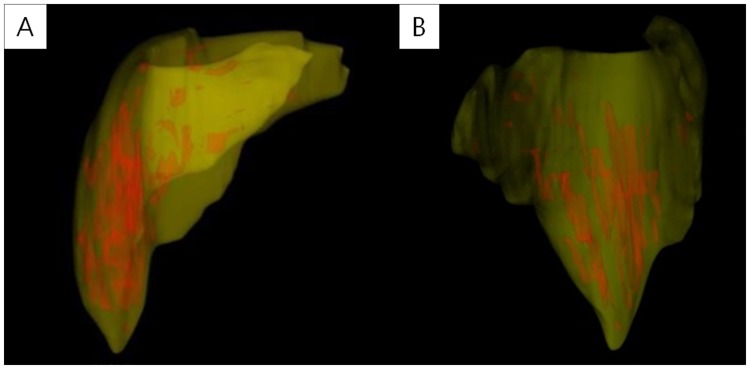
Generation of the 3-dimensional deltoid muscle model. (A) Anteroposterior and (B) lateral views of the 3-dimensional deltoid muscle model are shown. The deltoid muscle volume was computed automatically using this model.

The deltoid muscle volumes were measured using a picture archiving and communication system (PACS) software (Impax; Agfa, Antwerp, BE) with a 3-T Signa HDxt MRI scanner/Discovery MR750w system (General Electric, Milwaukee, WI, USA). The patient was positioned for imaging with the humerus in a neutral position and the thumb pointing upward. All MR images were obtained digitally using the PACS in the Digital Imaging and Communications in Medicine format. Imaging was performed from the supraclavicular portion down to the deltoid insertion portion using an axial T1-weighted turbo spin-echo technique (repetition time range/echo time range, 434–565/18–24; section thickness, 3 mm; field of view, 15 cm; matrix size, 224 × 224). The Mimics v17 medical imaging processing software (Materialise, Leuven, Belgium) was used to determine the 3-dimensional deltoid muscle volume. For the segmentation of the deltoid muscle in 2-dimensional T1-weighted MR axial images, the following image processing steps were performed. First, the region of interest (ROI) comprising the whole deltoid muscle extent from the MR image was designated (Fig 2A). Second, the bone and fat areas were separated from the established ROI (Fig 2B). Third, the deltoid muscle area of the 2-dimensional axial MR image was demarcated and segmented in each slide (Fig 2C). Finally, the 3-dimensional deltoid muscle model was generated by summing up each segmented 2-dimensional deltoid muscle area (Figs 2D and 3).

After generating the 3D deltoid muscle model, the 3D volume of the model was automatically computed. The deltoid muscle volume was adjusted afterward for the patients’ BMI. Previous studies evaluated the 3-dimensional volume of the thigh and paraspinal muscle using a multiplying method of the cross-sectional areas with a similar technique. [8] [29] [30] In line with a previous study, the dependability of this technique is outstanding, with an intraobserver intraclass correlation (ICC) of 0.97 and an interobserver ICC of 0.77 [31]. To appraise the intraobserver and interobserver reliabilities of our series, professional medical imaging analysts (A.S. and J.K.J.) separately and randomly measured each deltoid muscle volume. Each rater was unaware of the other’s ratings. For intraobserver reliability, 1 rater (A.S.) performed a second measurement with the same images 3 weeks after the first measurement while being unaware of the initial rating in all patients. The intraobserver and interobserver agreements of the deltoid muscle volume measurements were generally outstanding with ICCs of 0.947 for intraobserver reliability (p<0.001) and 0.846 for interobserver reliability (p = 0.017).

### Surgical procedures

All procedures were performed using an anterior delto-pectoral approach with the patient in the beach chair position. The prostheses inserted in all patients included the Aequalis reverse arthroplasty system (Tornier, Montbonnot Saint Martin, FR) with a 155° neck shaft angle.

During the surgical approach, the remnant tendinous portion of the subscapularis was detached from the insertion area of the proximal humerus. The glenohumeral joint was approached; the labrum was entirely eliminated; and the glenoid was totally exposed. The glenoid component and humeral stem were implanted in accordance to the guidelines provided by the manufacturer. The joint cartilage and bone of the glenoid side were removed by a manufacturer’s reamer, and the baseplate was placed with care roughly inferior to the center of the glenoid with inferior inclination to prevent an inferior notching phenomenon. [32] Humeral cutting was also carried out with an adequate retroversion between 0° and 20° subsequent to eliminating all spurs from the proximal humerus, and the stem and component for the humerus were inserted with an antibiotics (gentamicin) laden bone cement. Ahead of the final implantation, adequate tension, stability of the implant, and no impingement during ROM were appraised. Other combined additional surgeries (including the glenoid bone graft and transfer of the latissimus dorsi) were not carried out in all patients. All patients underwent similar postoperative protocols with an abduction brace for 1 month. Assisted ROM exercise was started after weaning off the brace. Strengthening program of the shoulder and periscapular muscle began 3–4 months weeks following the index operation.

### Outcome assessment

All patients underwent clinical and radiographic evaluations. The clinical appraisals were performed using shoulder scoring systems and active shoulder ROM preoperatively and postoperatively. Forward elevation was quantified in degrees between the arm and the thorax, with the elbow held straight. External rotation at the side was quantified in degrees between the thorax and the forearm, with the arm held in an adducted position and the elbow in 90° flexion. Internal rotation of the shoulder was quantified by the spine level reached at the back with the tip of the thumb. The vertebral level of the rotation was numbered serially as follows: 1 to 12 for the 1^st^ to 12^th^ thoracic vertebrae, 13 to 17 for the 1^st^ to 5^th^ lumbar vertebrae, and 18 for any level below the sacral region. The primary outcome parameters were set as age- and sex-matched Constant scores (above mean vs. below mean values) [26]. Patients who had a higher score compared to the average grounded on age- and sex-matched Constant scores [33] were categorized as the good function group, whereas those with a lower score than the average as the poor function group.

### Statistics

The dependability of the measurements of each deltoid muscle volume was appraised by examining the intraobserver and interobserver agreements determined using the ICC, which is a two-way random model with an absolute agreement. A paired t-test was used to compare the preoperative and postoperative results of pain using the VAS, ROM, and functional scores. The mean values were compared using the Mann-Whitney U test for continuous variables and the χ^2^ test or Fisher’s exact test for categorical variables to determine the differences between the good function and poor function groups. Pearson correlation analysis was used for correlation analysis between deltoid volume and clinical factors. Furthermore, a multivariate logistic regression analysis was used to determine the independent factors that influence the practical consequences by inputting the significant variables derived from the univariate analysis using the stepwise forward conditional method. The SPSS 13.0 (SPSS, Inc., Chicago, IL, USA) was used for all statistical analyses, and a p<0.05 was considered statistically significant.

## Results

All practical consequences comprising pain VAS, active forward flexion and external rotations, and diverse outcome measurements considerably enhanced following surgery (all p<0.01) (Table 2).

**Table 2:** Functional outcomes after reverse total shoulder arthroplasty.

Variables	Preoperative	12 months Postoperative	p-value
Pain VAS	6.25±2.24	2.97±1.97	***< 0*.*001***
ASES score	41.91±17.24	71.83±24.0	***< 0*.*001***
Constant score*	42.59±18.34	74.75±17.74	***< 0*.*001***
SST score	2.82±2.13	7.31±2.45	***< 0*.*001***
Forward elevation (°)	68.28±33.45	132.71±25.36	***< 0*.*001***
External rotation (°)	23.34±16.09	36.57±19.69	**0.004**
Internal rotation (vertebra) ^†^	13.31±3.81	11.89±3.14	0.082

VAS, visual analog scale; ASES, American Shoulder and Elbow Surgeons; SST, simple shoulder test

*Constant score was matched according to age and sex.[34]

^†^The vertebral level of internal rotation was numbered serially as follows: 1–12 for the 1^st^ to 12^th^ thoracic vertebra, 13–17 for the 1^st^ to 5^th^ lumbar vertebra, and 18 for any level below the sacral region.

In correlation analysis, deltoid volume is significantly correlated with Constant score [r (correlation coefficient) = 0.525, p = 0.001], forward flexion (r = 0.402, p = 0.017), and external rotation (r = 0.334, p = 0.050) (Table 3).

**Table 3:** The result of the correlation analyses between deltoid muscle volume and various clinical outcomes.

Variables	Correlation Coefficient	P-value
Pain VAS	-0.254	0.141
ASES score	0.205	0.237
Constant score*	0.525	***0*.*001***
SST score	0.252	0.144
Forward elevation (°)	0.402	***0*.*017***
External rotation (°)	0.334	***0*.*050***
Internal rotation (vertebra) ^†^	-0.294	0.087

VAS, visual analogue scale; ASES, American Shoulder and Elbow Surgeons; SST, simple shoulder test

*Constant score was matched according to age and sex.[34]

^†^The vertebral level of internal rotation was numbered serially as follows: 1–12 for the 1^st^ to 12^th^ thoracic vertebra, 13–17 for the 1^st^ to 5^th^ lumbar vertebra, and 18 for any level below the sacral region.

Overall, 20 showed a higher practical consequence compared to the average, while 15 showed a lower practical consequence, which was assessed by the age- and sex-matched Constant scores. The total deltoid volume adjusted for BMI (p = 0.009), absence of a subscapularis complete tear (p = 0.040), and greater change of the acromion-deltoid tuberosity distance (p = 0.013) were associated with higher matched Constant scores (Table 4).

**Table 4:** Comparison between the good function and poor function groups.

Variables	Good function group^∬^ (n = 20)	Poor function group^∬^ (n = 15)	p-value
Age (years)	74.20±3.62	75.53±4.95	0.364
Gender (n)	Male, 5; Female, 15	Male, 3; Female, 12	>0.999
Symptom duration (months)	39.65±56.19	73.53±90.96	0.183
Etiology (n)	CTA, 14; IMT, 6	CTA, 11; IMT, 4	>0.999
Side of involvement (n)	D, 16; ND, 4	D, 13; ND, 2	0.680
Body mass index (kg/m^2^)	25.49±3.11	25.89±2.60	0.695
Diabetes (n)	Yes, 5; No, 15	Yes, 5; No, 10	0.712
Hypertension or any heart disease (n)	Yes, 9; No, 11	Yes, 7; No, 8	>0.999
Bone mineral density	-1.76±1.29	-2.29±1.28	0.240
Shoulder usage level (n)	H, 4; M, 8; L, 8	H, 5; M, 3; L, 7	0.412
*Subscapularis integrity (n)	Intact or PT, 17; CT, 3	Intact or PT, 8; CT, 7	***0*.*040***
Pseudoparalysis (n)	Yes, 18; No, 2	Yes, 11; No, 4	0.367
^†^Fatty infiltration of the supraspinatus	2.85±0.93	3.33±0.81	0.120
^†^Fatty infiltration of the infraspinatus	2.85 ± 0.98	2.93±1.16	0.820
^†^Fatty infiltration of the subscapularis	2.00±1.25	2.40±1.12	0.337
^†^Fatty infiltration of the teres minor	1.50±1.14	2.06±1.38	0.195
*Volume of the total deltoid (mm^2^)/BMI (kg/m^3^)	4471.18±1902.32	2913.39±1185.85	**0.009**
Massive tear grade by Hamada classification (G1:G2:G3:G4a:G4b:G5; n)	3:7:1:5:3:1	1:3:2:3:4:2	0.660
Postoperative notching (n)	Yes, 10; No, 10	Yes, 10; No, 5	0.492
Notching grade by Sirveaux classification (G0:G1:G2:G3:G4; n)	10:6:2:2:0	5:5:5:0:0	0.220
Inferior overhang (n)	Yes, 18; No, 2	Yes, 13; No, 2	>0.999
Distance of inferior overhang (mm)	5.57±2.85	4.37±3.24	0.254
Glenosphere version (°)	97.91±11.41	103.08±8.06	0.145
Glenosphere-scapular neck angle (°)	94.75±12.42	95.24±6.35	0.935
*Change of the acromion-DT distance (mm)	32.01±12.21	20.38±8.41	***0*.*013***
Change of the COR distance (mm)	20.91±4.90	20.01±6.39	0.636
Preoperative pain VAS	5.95±1.98	6.67±2.55	0.357
Preoperative ASES score	42.35±14.89	41.32±20.49	0.865
Preoperative Constant score^‡^	41.45±19.44	38.33±18.84	0.638
Preoperative SST score	2.60±1.67	3.13±2.67	0.473
Preoperative Forward elevation (°)	66.00±32.83	71.33±35.17	0.648
Preoperative External rotation (°)	24.85 ± 14.10	21.33 ± 18.75	0.530
Preoperative Internal rotation (vertebra) ^§^	13.90 ± 3.41	12.53 ± 4.29	0.302
Postoperative pain VAS	2.75±1.90	3.20±2.13	0.742
Postoperative ASES score	78.56±18.61	62.87±27.93	0.054
Postoperative Constant score^‡^	85.90±5.45	58.20±10.49	<0.001
Postoperative SST score	8.50±1.82	5.73±2.34	<0.001
Postoperative Forward elevation (°)	148.50±16.31	111.67±19.24	<0.001
Postoperative External rotation (°)	50.05 ± 11.79	18.01 ± 10.31	<0.001
Postoperative Internal rotation (vertebra) ^§^	10.95 ± 2.78	13.13 ± 3.24	0.040

*Statistically significant

^†^Fatty infiltration was graded according to the criteria by Goutallier et al.[22]

^‡^Constant score was matched according to age and sex.[34]

^§^The vertebral level of internal rotation was numbered serially as follows: 1–12 for the 1^st^ to 12^th^ thoracic vertebra, 13–17 for the 1^st^ to 5^th^ lumbar vertebra, and 18 for any level below the sacral region.

^∬^Good function group included those who showed high postoperative matched Constant scores (above average), and poor function group included those with low postoperative matched Constant scores (below average).

CTA, cuff tear arthropathy; IMT, irreparable massive tear; D, dominant; ND, non-dominant; H, high level; M, middle level; L, low level; PT, partial tear; CT, complete tear; BMI, body mass index; DT, deltoid tuberosity; COR, center of rotation; VAS, visual analogue scale; ASES, American Shoulder and Elbow Surgeons; SST, simple shoulder test.

Other factors, such as the grade of the massive cuff tear, FD of the rotator cuffs, and other various radiographic parameters, were not associated with the practical consequences. Multivariate analysis indicated that the deltoid muscle volume was the single independent prognostic factor for the practical consequences (p = 0.011) (Table 5).

**Table 5:** Factors affecting the functional outcome after RTSA: The result of multivariate logistic regression analysis (Stepwise forward: Conditional).

Variable	p-value	Exp (B)	95% CI
*Deltoid muscle volume/BMI	0.011	1.126	1.028–1.233
Change of the acromion-DT distance^†^	0.079		
Subscapularis integrity^†^	0.153		

*Statistically significant.

^†^Variables not significant in multivariate analysis (those exempted from the equation).

Functional outcome was assessed by the age- and sex-matched Constant score.[34]

Postoperative scapular notching was noted in 20 (57.1%) patients, without influencing the practical consequences. One patient had an acromioclavicular separation 6 months postoperatively, and 1 had an acromial fracture 3 months postoperatively. Other complications involving dislocation or infection were not observed.

## Discussion

In this study, we explained the correlation between preoperative deltoid muscle volume and postoperative practical consequences following RTSA in patients with CTA or irreparable rotator cuff tears. Age- and sex-matched Constant scores were employed as the practical consequence measurement. As the Constant score covers the clinically most relevant domains and shows high responsiveness, and has been reported to be the most objective shoulder functional outcome instrument, with a total of 65 points for objective measures compared with other instruments, we selected this tool. [34,35] In this study, higher deltoid volumes, absence of a complete subscapularis tear, and greater change in the acromion-deltoid tuberosity distance were associated with the postoperative practical consequences in the univariate analyses. Among these, the deltoid muscle volume was the single independent prognostic factor for the practical consequences, which is the main finding of this study.

To our knowledge, Greiner et al. [36] were the first to determine the correlation between deltoid muscle status and practical consequences after RTSA. They revealed that the degree of postoperative FD of the deltoid is negatively correlated with the postoperative Constant score [36]; however, it was limited to a qualitative assessment of the deltoid. The quantitative measurement of the deltoid muscle area was first introduced by Meyer et al. [37] They measured the deltoid muscle area on T2-weighted fat-suppressed axial MR images at the mid-glenoid level to evaluate the correlation between the deltoid muscle area and shoulder function in patients with chronic rotator cuff tears. Nevertheless, measuring the 2-dimensional deltoid muscle area in a single image cut is insufficient to estimate the amount of muscle mass and may overlook vital morphologic information. [29] In addition, the comparison of exact MRI scan slices between 2 scans may not be feasible owing to the slice thickness. Therefore, we evaluated the 3-dimensional volume of the deltoid muscle to overcome the limitations of the qualitative FD measurement and the 2-dimensional assessment of a single image by using the multiplying method of the cross-sectional areas according to the previous technique used for the thigh and paraspinal muscle volumes [8] [31] [30]. The results revealed that the deltoid muscle volume was fairly associated with the practical consequences assessed by matched Constant scores. We believe that the muscle volume assessments, rather than the qualitative FD measurements or the cross-sectional area assessments, provide the true status of the deltoid muscle atrophy and muscle strength.

The correlation between a larger muscle volume and higher muscle strength and function has been well demonstrated previously [8] [30] [38] [39] [40]. Lindemann et al. [8] reported that thigh muscle volume is highly predictable for a sit-to-stand performance power. Similarly, Schantz et al. [7] reported that maximal torque correlates strongly with the muscle cross-sectional area. In addition, Bamman et al. [41] revealed that the MRI-determined triceps surae muscle size indices correlate with strength better than the whole limb anthropometric and dual energy X-ray absorptiometry indices. Thus, the larger volume of the deltoid may indicate the stronger deltoid in this study. Given that the RTSA depends mostly on the deltoid as a substitute for a nonfunctioning rotator cuff, we believe that the larger volume or the stronger deltoid may facilitate the operation of the reverse prosthesis, thus improving the practical consequences. We can also find a likeness of the key muscle strength with the practical consequences following arthroplasty, such as between the quadriceps muscle and total knee arthroplasty or between hip abductor muscle and total hip arthroplasty. Mizner et al. [42] suggested that preoperative quadriceps strength plays a dominant role in predicting practical consequences following total knee arthroplasty. Sathappan et al. [43] demonstrated that hip abductor muscle weakness is related to surgical consequences following total hip arthroplasty. Thus, anticipating good functional outcomes after RTSA seems reasonable, where the deltoid function is fundamental in patients with bigger and stronger deltoids. Several authors highlighted the importance of the deltoid muscle for shoulder function and surgical consequences of RTSA. Ackland et al. [44] reported that the deltoid is the most effective flexor and has substantial potential to initiate flexion after RTSA. Furthermore, Whatley et al. [45] also underlined the importance of the deltoid; they showed that deltoid rupture following RTSA leads to poor practical consequences. Thus, the deltoid muscle volume and strength exercises, such as preoperative deltoid strengthening exercises, should be performed particularly in patients with a small and weak deltoid who are scheduled to undergo RTSA to improve practical consequences following RTSA. Tungtrongjit et al. [46] support the possible benefit of deltoid strengthening exercises in patients who underwent RTSA. They demonstrated that preoperative quadriceps exercise at least 3 weeks prior to total knee arthroplasty decreases pain and improves practical consequences.

To our knowledge, this study is the first to perform quantitative measurement of the deltoid volume in a somewhat homogeneous group of irreparable massive rotator cuff tears or cuff-tear arthropathy and evaluate its relationship with the practical consequences following RTSA.

However, there were several limitations. First, the number of patients in our study was relatively small, which could be the reason for the lack of statistical significance in some factors. The post hoc power analysis indicated that we had only a 58.6% power to detect a 10.4 point difference for Constant score which was previously shown to be a minimal meaningful clinical difference for Constant score in patients undergoing shoulder surgery,[47] in the 35 patients with an alpha error of 0.05. Further studies with larger number of cases may be required for excluding the probability of type 2 errors. Second, a measurement error in the deltoid muscle volume and other radiographic parameters may have occurred. However, as the interobserver and intraobserver reliabilities of the measurements of each deltoid muscle volume were excellent in this study, we believe that the measurement error was minimal, if any. However, as high number of patients had notching in our series, the landmarks for measuring overhang and medialization of center of rotation cannot be accurate. Thus, there could be an inevitable measurement error for the radiographic parameters. Finally, the clinical period for follow-up was insufficient. The practical consequences may change further in a longer follow-up period. This study may cover the results of short to intermediate-term follow-ups.

## Conclusions

The practical consequences after reverse shoulder arthroplasty in patients with CTA or irreparable rotator cuff tears were considerably impacted by the preoperative deltoid muscle volume. Therefore, more attention should be paid to patients with severe atrophied deltoid muscle who are at a high risk for poor practical consequences after RTSA.

## Supporting information

S1 File Minimal dataset of this study. (SAV)
